# Systematic Identification of Multi Omics-based Biomarkers in *KEAP1* Mutated TCGA Lung Adenocarcinoma

**DOI:** 10.7150/jca.35489

**Published:** 2019-11-01

**Authors:** Akhileshwar Namani, Zhaohong Zheng, Xiu Jun Wang, Xiuwen Tang

**Affiliations:** 1 Department of Thoracic Surgery and Department of Biochemistry of the First Affiliated Hospital, Zhejiang University School of Medicine, Hangzhou 310003, PR China;; 2 Department of Pharmacology, and Cancer Institute, The Second Affiliated Hospital, Zhejiang University School of Medicine, Hangzhou 310009, PR China.

**Keywords:** TCGA-Lung adenocarcinoma, KEAP1 mutations, NRF2 biomarkers, gene signature

## Abstract

Mutations in *KEAP1* and/or *NRF2* genes have been identified across many cancers and the dysregulation of the NRF2 pathway due to these mutations leads to drug and radioresistance in several cancers. Identification of biomarkers associated with these mutations allows the researchers and clinicians to identify the personalized medicine and quicker diagnosis. In this current study, we carried out an integrated, multi-omics, multi-database analysis of exome, transcriptomics data's of *KEAP1* mutated TCGA- Lung adenocarcinoma (LUAD) patients against non-mutated counterparts. Finally, we discovered the gene signature associated with *KEAP1* mutations, prognostic genes which were highly correlated with the upregulation of the NRF2 pathway in the KEAP1 mutated LUAD patients. Our finding might be useful to identify the early diagnosis of KEAP1 mutated LUAD patients.

## Introduction

Lung cancer reported as one of the highest cancer-related deaths worldwide which accounts for more than 1.2 million deaths annually 1. The most common subtype of lung cancer is Non-small cell lung cancer (NSCLC) attributed 85% of lung cancers and the overall 5-year survival rate is very low (16%) 2. Currently, personalized medicine and targeted therapies are available for a small subset of lung cancer patients 3. Emerging cancer genomics studies generated by the Cancer Genome Atlas (TCGA) have provided high throughput data of several cancers including the genetic landscape of Lung adenocarcinoma (LUAD) 4 which led the researchers to identify the underlying mechanisms of mutation-specific lung tumorigenesis.

Kelch-like ECH-associated protein 1 (KEAP1)/nuclear factor erythroid 2-related factor 2 (NFE2L2 or NRF2) pathway plays a major role in redox homeostasis. NRF2 combat against oxidative stress in mammalian cells during redox imbalance by inducing the expression of several cytoprotective genes 5. Under homeostatic conditions, KEAP1 negatively regulates the NRF2 *via* Cullin3 (CUL3) mediated ubiquitination followed by proteasomal degradation 6. Dysregulation of KEAP/NRF2 pathway due to loss of function mutations in the *KEAP1* gene as well as gain-of-function mutations in NRF2 and epigenetic changes leads to drug- and radio-resistance in lung cancer 7. The high frequency of *KEAP1* mutations has been considered as an important molecular event in lung cancer progressions 8. Seminal studies on NRF2 pathway revealed its specific role in metabolic reprogramming 9, carbon metabolism 10, serine biosynthesis 11 in NSCLC.

Multi-centered high throughput exome sequencing data of TCGA-LUAD tumors revealed the mutation landscape of several genes including KEAP1 mutations 4. In our previous studies, we discovered the gene signatures which are regulated by NRF2 pathway in NSCLC 12 and TCGA-head and neck squamous cell cancer (HNSCC) 13 and identified the prognostic effect of these genes in both cancers. In the current study, we utilized the genomics, and transcriptomics data of a LUAD cohort from TCGA study. Finally, we comprehensively identified the *KEAP1* mutation specific prognostic biomarkers and validated using qRT-PCR. These analyses allowed us to compile comprehensive transcriptomic profiling of mutational landscape associated with *KEAP1* gene.

## Materials and Methods

### Overall LinkedOmics database analysis

To specifically address the relationships between *KEAP1* somatic mutations and clinical outcomes in LUAD, we utilized multi-omics TCGA studies based database - 'LinkedOmics' 14 and analyzed the mutation, mRNA expression data of the KEAP1 mutated TCGA-LUAD tumors. The entire patient's omics data was used in linkedomics was obtained from the pre-processed data of the Broad Institute- Firehose Pipeline.

### RNA-Seq data analysis

Primarily, we focused on the identification of the *KEAP1* mutation associated differentially expressed genes (DEG's) from the RNA-Seq data of LUAD patients. Briefly, we selected TCGA-LUAD as our interested 'cancer cohort' followed by the selection of dataset (Exome sequencing data: n=533), search dataset attribute/gene (our gene of interest-KEAP1) and target dataset attribute (RNAseq: n=515-Illumina HiSeq platform) respectively. 478 samples were overlapped between search dataset attribute and target dataset attribute, of which 83 were *KEAP1* mutated and 395 were wild type (other than *KEAP1* mutated). We applied t-test and Wilcoxon tests to obtain KEAP1 associated DEG's. DEG's expression fold change (FC) cut off >1.5 were considered for the identification of *KEAP1* mutation-specific gene cluster (KMSGC). P<0.001 was used as the cutoff for significance. All the genes that have expression values in 478 samples were only considered for the DEG analysis. Heat map of KMSGC was created using 'Heatmapper' tool 15.

We also retrieved the publicly available RNA-Seq results of NRF2 knockdown (NRF2 KD) LUAD cell lines such as A549 from Olagnier *et al,* 2018 (GSE113519) 16 and H2122 from Bar-Peled *et al*, 2017 (GSE89569) 17 to cross check the expression pattern of KMSGC. To visualize the heat maps of KMSGC in both cell lines, we used the web-based RNA-Seq analysis tool- START App 18.

### Functional annotation analysis

WebGestalt (WEB-based Gene SeT AnaLysis Toolkit) web tool 19 was used to annotate the functional enrichment analysis of KMSGC obtained from the upregulated DEG's list. Among the different functional annotation types present in WebGestalt, the analysis was specifically focused on the GO biological processes and KEGG pathways.

### Position Weight Matrix (PWM) genome-wide NRF2 binding sites identification

*In silico* analysis of KMSGC was carried out by using the data retrieved from both PWM Scan 20 and ChIPSeek web tools 21. Firstly, we downloaded the matrix (Matrix ID: MA0150.1) encoding the NRF2-ARE from the transcription factor binding profiles database named JASPAR 22. Secondly, we generated the BED file using PWM Scan web tool for hg 19 version of the human genome. Finally, the generated BED file was uploaded for gene annotation using ChIP-Seq analysis tool known as ChIPSeek 21. In addition, we used the Encyclopedia of DNA Elements (ENCODE) consortium's NRF2 ChIP-Seq binding sites data from A549 cells 23 and publicly available NRF2-A549 cells ChIP-Seq data from Olagnier et al, 2018 19 (GSE113497) for comparative analysis.

### Cell cultures

A549 NSCLC cell line was from the American Type Culture Collection (ATCC, Beijing, China). A549-derived stable siRNA knockdown for NRF2 (siNrf2-C27) and, control cell line (siGFP-C5) were generated as described previously 24. The cells were maintained in a growth medium containing Dulbecco's MEM with Glutamax supplemented with 10% fetal bovine serum (FBS) and antibiotics. All cells were cultured at 37^o^C, in 95% air and 5% CO_2_, and passaged every 3 to 4 days. All medium supplements for cell culture were from Invitrogen (Shanghai, China).

### RNA isolation and qRT-PCR

Total RNA was prepared using TRIzol reagent (Invitrogen) and the qRT-PCR procedure was performed described previously 24. The primers used for the validation were obtained from primer bank 25 except AKR1C1, NQO1 24, and listed in Table S6. p values <0.05 were considered statistically significant.

### Survival analysis

For the identification of prognostic biomarkers, Kaplan-Meier curves were calculated and generated by using web-based patients survival analysis tool SurvExpress 26 for upregulated genes P<0.05 was used as the cutoff for significance. The method of analysis was discussed in our previous studies 12, 13.

## Results

### Identification of differentially expressed genes (DEG's) among *KEAP1* mutated and wild type (WT) TCGA LUAD patients

**Figure 1:**
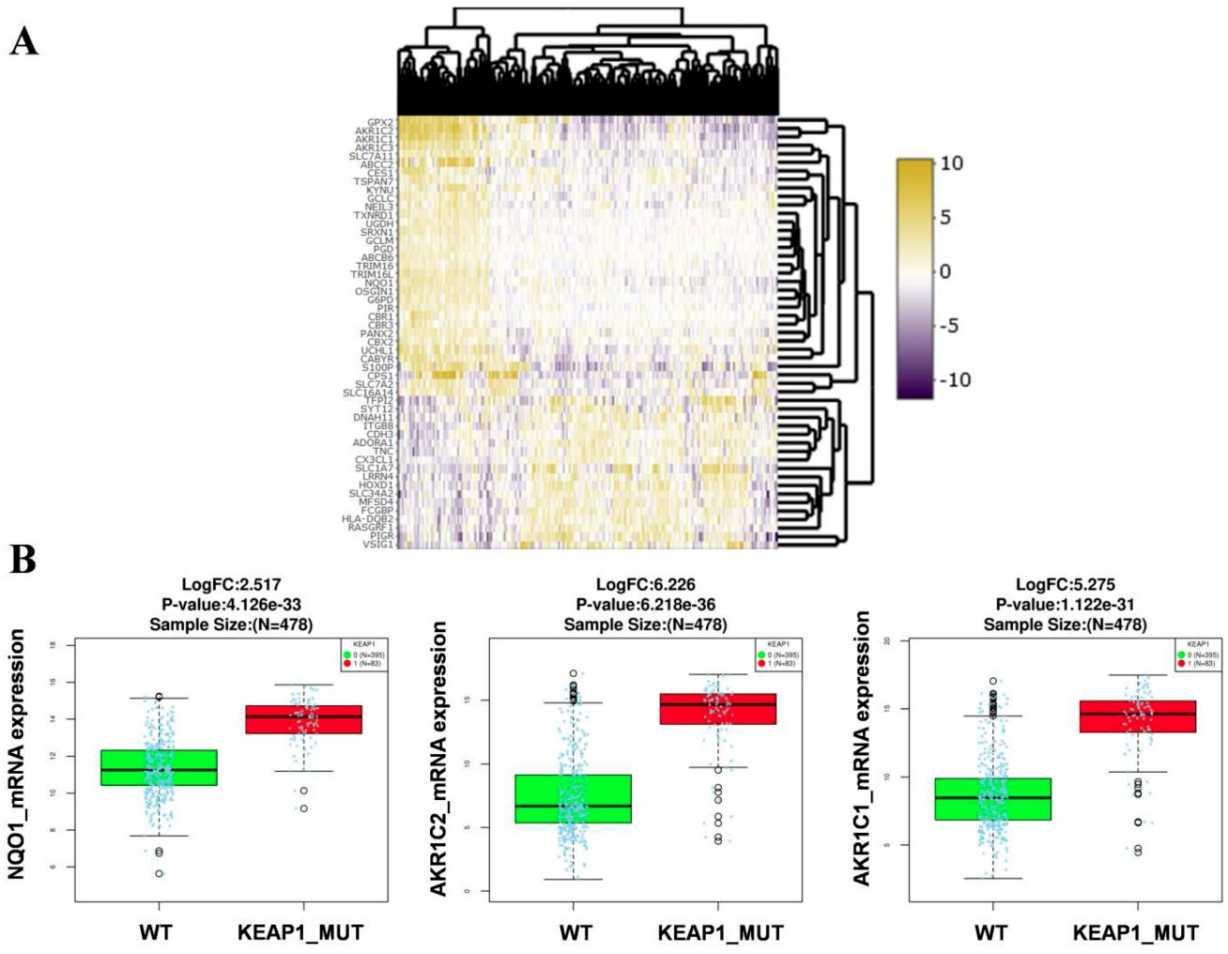
Identification of the differential expression of genes (DEG's) in KEAP1 mutated *versus* wild type TCGA-LUAD tumors. Heatmap showing the differential expression pattern of genes between KEAP1 mutated (KEAP1_MUT) and wild type (WT) TCGA-LUAD tumors, (B) box plots showing the higher expression of bonafide NRF2 target genes in KEAP1 mutated tumors as compared with WT tumors.

**Figure 2:**
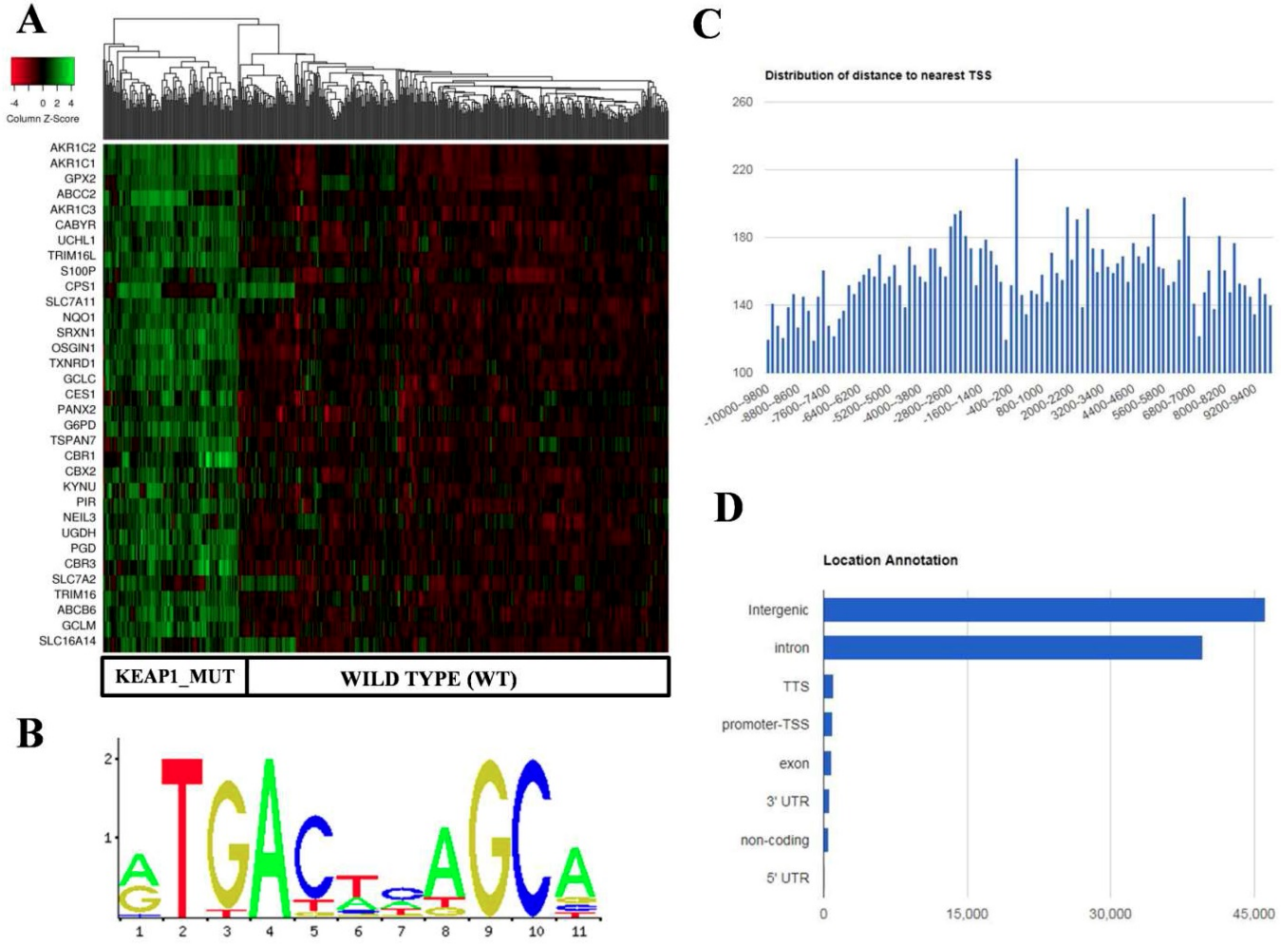
Identification and *in silico* analysis of KEAP1 Mutation Specific Gene Cluster (KMSGC) in TCGA-LUAD. (A) Heat map showing the overexpression of KMSGC in KEAP1 mutated LUAD tumors as compared with the WT counterparts. (B) Description of the NRF2-ARE JASPAR database matrix used for the PWM-Scan *in silico* analysis. (C) Distribution of the number of NRF2 binding sites within the 10 kb upstream and downstream of the promoter Transcription Starting Site (TSS). (D) Bar chart of the genomic location distribution of NRF2 binding sites obtained from ChIPseek tool. The X-axis shows the genomic location and Y-axis shows the number of NRF2 binding sites.

RNA-Seq gene expression data (Illumina HiSeq 2000 platform) of the TCGA-LUAD patients (478 samples) was examined to identify the genes and pathways associated with *KEAP1* mutations. Patient's barcodes were stratified into *KEAP1* mutant and wild type (WT) based on the mutational status (Table S1). Differentially expressed genes were identified using Linkedomics web tool 14 with a fold change of > 1.5 between *KEAP1* mutated and WT tumors by using both statistical tests such as t-test and Wilcoxon test with a stringent p-value cut off < 0.005 and considered the overlapping genes obtained from both tests to avoid false positive results. We then integrated the list of DEGs in both tests to obtain the overlapping genes by using 'venny' (Figure S1). As a result, we found 33 up and 18 downregulated overlapping genes (Table S2) in *KEAP1* mutated LUAD tumors. The differential expression analysis results in *KEAP1* mutated and WT tumors by using two statistical tests were displayed as a heatmap in Figure 1A. The upregulated genes list include several bonafide NRF2 target genes such as NQO1, AKR1C1, AKR1C2 (Figure 1B). Thus, the DEGs analysis results clearly show that the *KEAP1* mutations lead to the higher expression of NRF2 regulated genes in LUAD. Therefore, we focused on the 33 upregulated genes for further analysis and named these genes as *KEAP1* mutation specific gene cluster (KMSGC) (Figure 2A).

### *In silico* analysis identified the known and putative NRF2 binding sites in KMSGC

Previous studies including ChIP-Seq data revealed that NRF2 not only binds at the promoter regions of its target genes but also in the other regulatory regions of the genome 27. For instance, a recent study showed that NRF2 binds at the eighth intron of ABCC3 gene and regulates its gene expression 28. Among the 33 KMSGC, the majority of the genes possess well-characterized antioxidant responsive elements (AREs) in their promoter regions. However, the AREs which are located other than promoter regions of these genes remain unknown. To identify the putative and known AREs in the KMSGC, we utilized PWM Scan 20 and ChIPseek 21 web tools. Apart from *in silico* analysis, we employed The Encyclopedia of DNA elements (ENCODE) consortium NRF2-ChIP-Seq data in A549 cells 29 and publicly available NRF2-A549 ChIP-Seq data for the comparative analysis 16.

Using PWM Scan 20 and ChIPSeek web tool 21, a total of 89858 peaks encoding NRF2-binding sites (Figure 2B) were identified in the whole human genome (UCSC-hg 19 version). The genomic locations of total 89858 NRF2 binding sites which encode 19789 genes were annotated using ChIPSeek web tool (Table S3). The annotated genes showed a wide distribution pattern in which 15797 binding sites are present within 10 kb of TSS (Figure 2C). In total, 965 sites were present at proximal to the transcription start site (TSS) region, 39698 binding sites were in introns , 46164 were in intergenic, 827 were in exon , 82 were in 5' UTR, 993 were in TTS and 629 were found in 3' UTR region respectively (Figure 2D). This result shows that the majority of NRF2 binding sites were present in intergenic, followed by introns, exon, promoter-TSS and to a lesser degree in 3' UTR, TTS, non-coding and 5' UTR regions.

In the case of KMSGC binding patterns, we identified several NRF2 binding sites in the 28 genes among 33 KMSGC (Table S4). However, our *in silico* analysis didn't identify the NRF2 binding sites in the genomic sequences of 5 genes such as CBR1, CBR3, G6PD, PANX2, and S100P. Notably, our comparative transcription factor binding site (TFBS) analysis by using ENCODE 23 and Olagnier *et al,* 2018 16 NRF2-A549 ChIP-Seq data identified ARE's in the promoter regions of CBR1, CBR3, PANX2 genes except G6PD and S100P. Altogether, our *in silico* and comparative TFBS analysis suggesting that the majority of genes identified in KMSGC contain NRF2 binding sites and most of them are functionally active.

### KMSGC mRNA expression was highly downregulated in NRF2 knockdown NSCLC RNA-Seq data

To further confirm whether NRF2 regulates the KMSGC gene expression in lung adenocarcinoma, we used two publicly available RNA-Seq data of NRF2 knockdown (KD) NSCLC cells. RNA Seq FPKM values of two NRF2 KD lung adenocarcinoma cell lines such as A549 from Olagnier *et al,* 2018 (GSE113519) 16 and H2122 from Bar-Peled *et al*, 2017 (GSE89569) 17 studies were considered respectively. Interestingly, among the 33 KMSGC genes, 12 genes expression such as AKR1C1, NEIL3, GCLM, TRIM16L, OSGIN1, SRXN1, UGDH, TSPAN7, ABCB6, TXNRD1, PANX2, ABCC2 was significantly downregulated with the fold change (FC) >1.5 in both datasets. Whereas, CBR3, UCHL1, CBR1, CABYR, CBX2, PIR, GPX2 genes expression was specifically downregulated with FC>1.5 in NRF2 KD-H2122 cells. Likewise, SLC7A11, G6PD, TRIM16, AKR1C2, GCLC, NQO1, AKR1C3, PGD, CES1 were downregulated with FC >1.5 in NRF2 KD-A549 cells. Thus, out of 33 KMSGC, 28 genes were highly downregulated in NRF2 KD NSCLC cell lines (Figure S2). However, we didn't find significant gene expression changes of 5 genes such as CPS1, SLC16A14, SLC7A2, KYNU, and S100P in either of RNA-Seq data (Table S5). Altogether, our patients-specific KMSGC revealed that the majority of the genes are regulated by NRF2 in LUAD.

### Validation of novel NRF2 target genes in NSCLC cell lines

**Figure 3:**
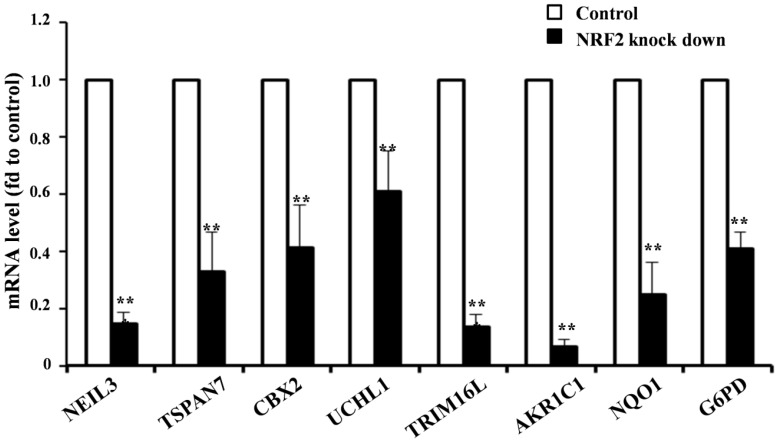
qRT-PCR analysis shows significantly decreased mRNA expression level in NRF2 KD cells as compared with control A549 cells. (*p < 0.05; ** p < 0.01, *** p < 0.001).

Given the higher expression of KMSGC in *KEAP1* mutated patients and NRF2 knockdown RNA-Seq data, we hypothesized that NRF2 directly transactivates the novel and known genes present in KMSGC. To evaluate whether *in silico* analyzed novel genes directly regulated by the NRF2 transactivation, we selected the five novel NRF2 regulated genes in KMSGC such as NEIL3, TSPAN7, CBX2, UCHL1, and TRIM16L along with bonafide NRF2 genes-AKR1C1, NQO1, G6PD and performed qRT PCR analysis in NRF2 knocked down A549 NSCLC cells as described previously 12. As shown in figure 3, NRF2 knockdown in A549 NSCLC cells exhibited significant downregulation of the selected genes. Thus, the putative* in silico* identified genes are regulated by NRF2 in lung adenocarcinoma.

### KMSGC is enriched in different metabolic pathways

We next utilized the WebGestalt 19 tool to perform functional annotation analysis of KMSGC. Interestingly, Gene Ontology results (GO slim classification) - Biological process identified the genes involved in the metabolic process, response to stimulus, multicellular organismal process, developmental process and others (Figure S3). In detailed, majority of the genes present in the biological processes such as response to oxidative stress, quinone metabolic process, response to toxic substance, cofactor metabolic process, response to acid chemical, cellular ketone metabolic process, detoxification, cellular response to acid chemical, secondary metabolic process, and response to nutrient levels (Table 1). We then carried out the KEGG pathway analysis by using the same tool to know the important pathways associated with KMSGC (Table 2). As anticipated, KEGG pathway analysis identified well-known NRF2 regulated pathways such as Glutathione metabolism, Arachidonic acid metabolism, Steroid hormone biosynthesis, Metabolism of xenobiotics by cytochrome P450, Pentose phosphate pathway, Carbon metabolism, Cysteine and methionine metabolism, ABC transporters, and Chemical carcinogenesis. Thus functional annotation results revealed that the genes listed in the KMSGC functionally related to NRF2 mediated drug metabolism and metabolic reprogramming in LUAD.

### The prognostic power of top *KEAP1* Mutation Associated Gene Signature (KMAGS) in LUAD

**Figure 4:**
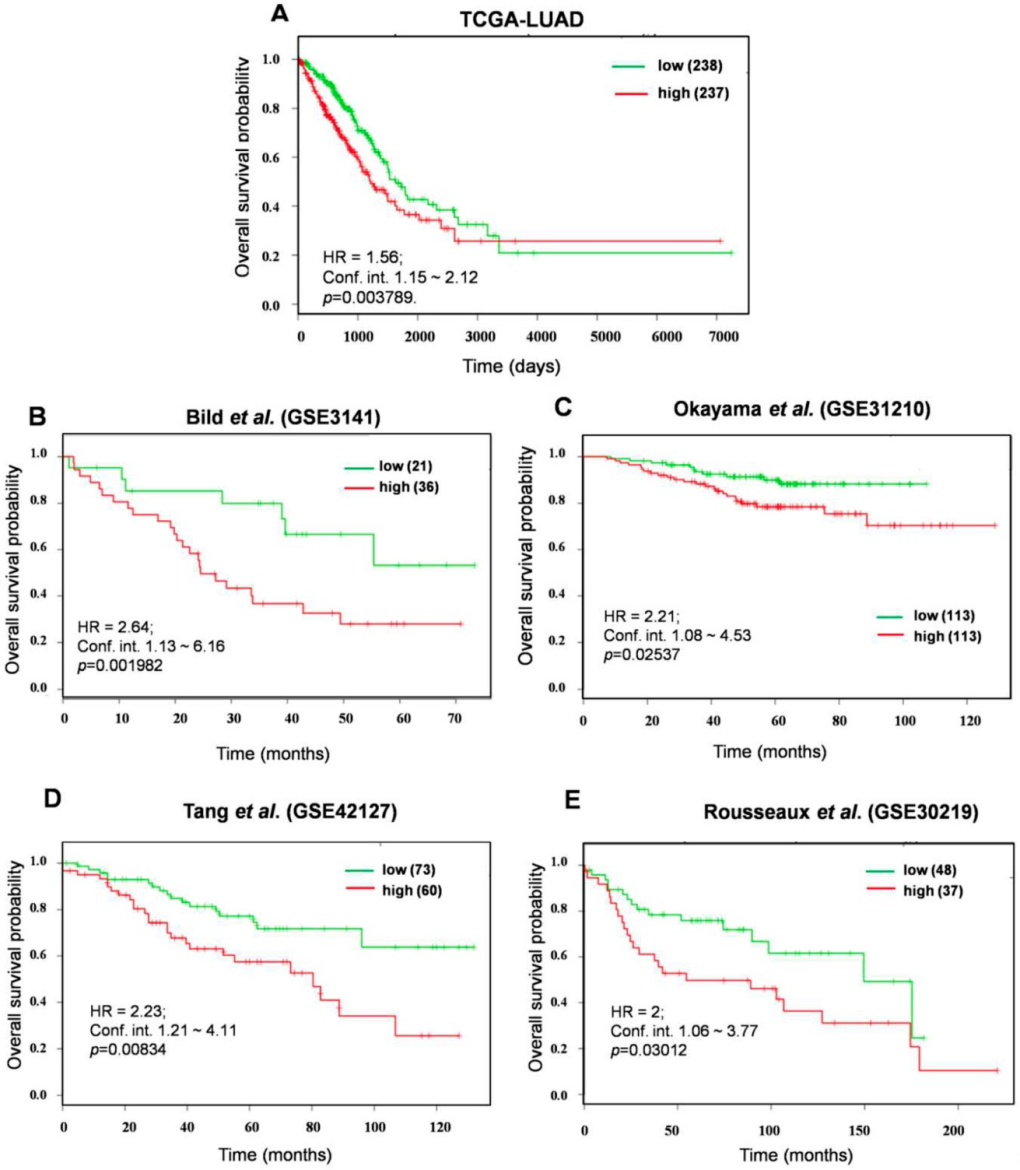
Prognosis prediction of KMAGS TCGA-LUAD. Kaplan-Meier plot shows that KMAGS overexpressed high-risk group of patients' shows the poor survival (p < 0.05) in TCGA-LUAD cohort. The high (red) and low (green) risk group of TCGA-LUAD patients was stratified based on the expression pattern.

In an effort to identify the prognostic biomarkers of KMSGC in LUAD, the survival analysis web tool-SurvExpress 26 was employed as described previously 12. It is important to consider a relatively small number of genes than the more number of genes for prognosis analysis 30, 31. For this analysis, we minimized the number of genes and strictly considered the 12 KMSGC genes among 33 genes which showed >2.5 fold higher expression in *KEAP1* altered patients as compared with Wild type patients data. This high expression of rank-based survival analysis would perfectly predict the NRF2 activation status in LUAD patients. We named these 12 genes as a *KEAP1* Mutation Associated Gene Signature (KMAGS) in LUAD. The 12 KMAGS include genes such as AKR1C2, AKR1C1, GPX2, ABCC2, AKR1C3, CABYR, UCHL1, TRIM16L, S100P, CPS1, SLC7A11, and NQO1 (Table S2). We first selected the parent cohort TCGA-LUAD (n=475) for the overall survival analysis by using SurvExpress 26. Notably, elevated expression of KMAGS associated with significantly poor survival in LUAD patients (Figure 4A). In addition to the parent cohort, we also performed overall survival analysis in other individual LUAD cohorts such as Bild et al. (GSE3141)^32^, Okayama et al. (GSE31210) 33, 34, Tang et al. (GSE42127) 35, and Rousseaux et al. (GSE30219) 36. As a result, we also found that higher expression of KMAGS leads to poor survival in LUAD patients (Figure 4 B-E). This result further supports the robust prognostic power of the KMAGS in LUAD.

## Discussion

Utilization of the different TCGA datasets to identify the molecular changes associated with specific gene mutations which are linked with the clinical outcomes has been demonstrated in many studies including lung cancer 12. Majority of the TCGA based studies have been focused on the identification of new prognostic markers and novel therapeutic targets in different cancers 13. Stratification of LUAD patients with *KEAP1* mutations provides us the clues to identify the discovery of personalized/precision medicine for the treatment. In the present study, we stratified the LUAD patient's samples as two groups named *KEAP1* mutant and WT and identified *KEAP1* mutation-specific prognostic gene signature which is associated with poor survival in LUAD patients. One of the major advantages of our study is that we performed the systematic analysis of TCGA-LUAD dataset which contains 478 patient's transcriptomics data and is by far the largest dataset for LUAD survival prediction.

Based on the DEG analysis of *KEAP1* mutated *versus* WT LUAD tumors, we identified 33 upregulated genes cluster whose expression is highly correlated with the *KEAP1* mutated patients. *KEAP1* Mutation Specific Gene Cluster (KMSGC) functional enrichment of genes in LUAD patient's shows highly activated metabolic pathways such as Glutathione metabolism, Pentose phosphate pathway (PPP) and Carbon metabolism. It has been well established that, NRF2 regulates the carbon metabolism and metabolic reprogramming in NSCLC 9. Transcriptomics analysis clearly shows that the *KEAP1* mutations in LUAD lead to the loss of function of *KEAP1* and gain of function of NRF2. For instance, mRNA and protein levels of key metabolic NRF2 regulated gene-GDPD highly upregulated in *KEAP1* mutant patients and elevated expression of G6PD could be used as a biomarker for the detection of NRF2 overexpression in LUAD. Further *in silico* study on NRF2 binding sites in our study revealed that the majority of the genes upregulated in *KEAP1* mutated tumors are regulated by the NRF2 through transactivation mechanism except for 2 genes such as G6PD and S100P. However, our literature survey found that G6PD is a direct target of NRF2 in NSCLC and possess functional ARE within 1kb to TSS 9. Interestingly, Chien *et al.* 2015 found that S100P was highly downregulated in both KEAP1 overexpressing and NRF2 KD NSCLC cells and promotes cell migration in NSCLC. Apart from known NRF2 target genes in KMSGC, we found novel NRF2 target genes such as TRIM16L, NEIL3, CBX2, and UCHL1 which contains ARE sequences in different genomic locations. Notably, our recent study on TCGA- head and neck cancer also identified the overexpression of TRIM16L and UCHL1 in KEAP1-NRF2-CUL3 axis altered patients. Altogether, our study suggests that genes present in the KMSGC could be important therapeutic targets for *KEAP1* mutated LUAD patient's treatment. In conclusion, by utilizing a large TCGA-LUAD patient cohort, our study identified a*KEAP1* mutation-specific gene signature, prognostic genes, which can be used as potential prognostic biomarkers for LUAD and also potential therapeutic targets.

## Supplementary Material

Supplementary figures and tables.

Supplementary table S3.

## Figures and Tables

**Table 1:** List of GO-biological processes associated with KMAGS

Gene set	Description	Overlap Gene ID	P-value	FDR
GO:0006979	Response to oxidative stress	ABCC2;SRXN1;NQO1;SLC7A11;G6PD;GCLC;GCLM;GPX2;TXNRD1;AKR1C3	1.55E-09	5.82E-06
GO:1901661	Quinone metabolic process	AKR1C1;AKR1C2;AKR1C3;CBR1;CBR3	3.49E-09	5.82E-06
GO:0009636	Response to toxic substance	CES1;ABCC2;CPS1;SRXN1;NQO1;SLC7A11;GPX2;TXNRD1	4.30E-09	5.82E-06
GO:0051186	Cofactor metabolic process	ABCB6;AKR1C1;AKR1C2;G6PD;PGD;AKR1C3;CBR1;CBR3;KYNU	1.88E-08	1.91E-05
GO:0001101	Response to acid chemical	TRIM16;ABCC2;CPS1;AKR1C1;AKR1C2;GCLC;GCLM;AKR1C3	9.26E-08	7.51E-05
GO:0042180	Cellular ketone metabolic process	AKR1C1;AKR1C2;NQO1;AKR1C3;CBR1;CBR3;KYNU	1.76E-07	0.000118657
GO:0098754	Detoxification	ABCC2;SRXN1;NQO1;GPX2;TXNRD1	9.72E-07	0.00056323
GO:0071229	Cellular response to acid chemical	CPS1;AKR1C1;AKR1C2;GCLC;GCLM;AKR1C3	1.32E-06	0.000668037
GO:0019748	Secondary metabolic process	ABCC2;AKR1C1;AKR1C2;AKR1C3	3.71E-06	0.001669786
GO:0031667	Response to nutrient levels	CPS1;NQO1;G6PD;GCLC;GCLM;AKR1C3;KYNU	7.53E-06	0.003052302

**Table 2:** List of KEGG pathways associated with KMAGS

KEGG pathway	Overlap Gene ID	P-Value	FDR
			
hsa00480-Glutathione metabolism	G6PD;GCLC;GCLM;GPX2;PGD	3.26E-07	9.06E-05
hsa00590-Arachidonic acid metabolism	GPX2;AKR1C3;CBR1;CBR3	2.46E-05	0.003419094
hsa00140-Steroid hormone biosynthesis	AKR1C1;AKR1C2;AKR1C3	0.000561716	0.052052368
hsa00980-Metabolism of xenobiotics by cytochrome P450	AKR1C1;CBR1;CBR3	0.001146066	0.079651589
hsa00030-Pentose phosphate pathway	G6PD;PGD	0.003234795	0.179854598
hsa01200-Carbon metabolism	CPS1;G6PD;PGD	0.003951649	0.183093065
hsa00270-Cysteine and methionine metabolism	GCLC;GCLM	0.007175004	0.249331393
hsa02010-ABC transporters	ABCB6;ABCC2	0.007175004	0.249331393
hsa05204-Chemical carcinogenesis	AKR1C2;CBR1	0.022593593	0.697890973
			

## Abbreviations

ARE: antioxidant responsive elements
CUL3: cullin3
DEG: differentially expressed genes
ENCODE: The Encyclopedia of DNA elements
HNSCC: head and neck squamous cell cancer
KEAP1: Kelch-like ECH-associated protein 1
KMAGS: KEAP1 mutation associated gene signature
KMSGC: KEAP1 mutation specific gene cluster
LUAD: lung adenocarcinoma
NRF2: nuclear factor erythroid 2- related factor 2
NSCLC: non- small cell lung cancer
PWM: position weight matrix
TCGA: The Cancer Genome Atlas
