# Supplementary material for: Systematic Identification of Multi Omics-based Biomarkers in KEAP1 Mutated TCGA Lung Adenocarcinoma
Source: J Cancer. 2019 Nov 1;10(27):6813–21. doi: 10.7150/jca.35489 (PMC6909944; doi:10.7150/jca.35489)
Supplement: Supplementary file 1 — Supplementary figures and tables. [file jcav10p6813s1.pdf]

Supplementary figures

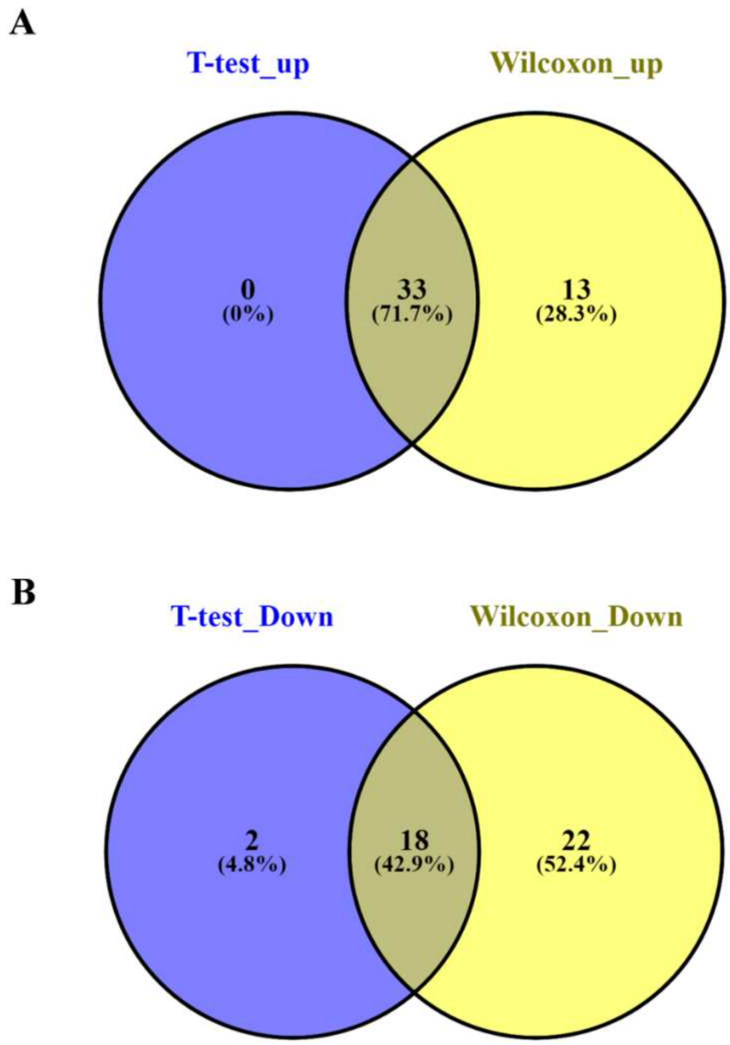

Figure S1. Integrated analysis of up (A) and down regulated (B) genes obtained from the KEAP1 mutation versus WT LUAD tumors.

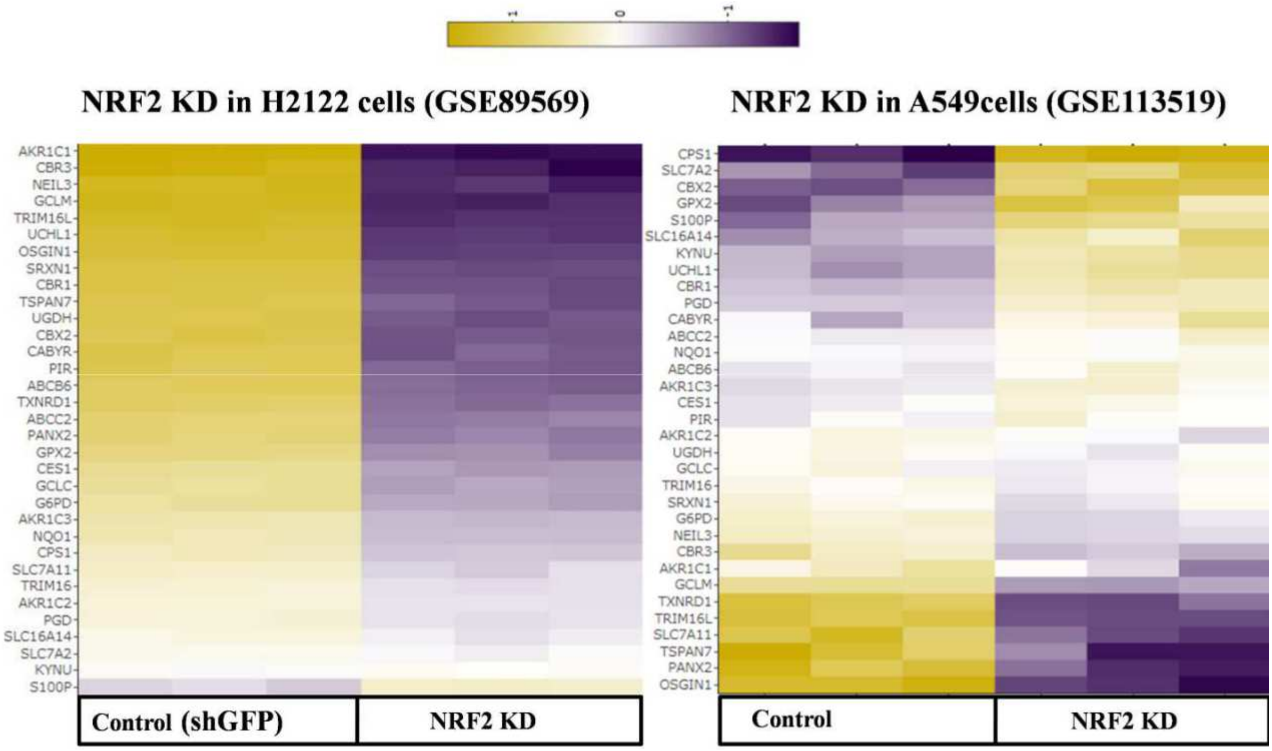

Figure S2. Patterns of KMSGC gene expression in RNA-Seq data of NRF2 knockdown lung adenocarcinoma cell lines such as H2122 from (A) Bar-Peled *et al*, 2017 (GSE89569) [16] and (B) A549 from Olagnier *et al*, 2018 (GSE113519) [15].

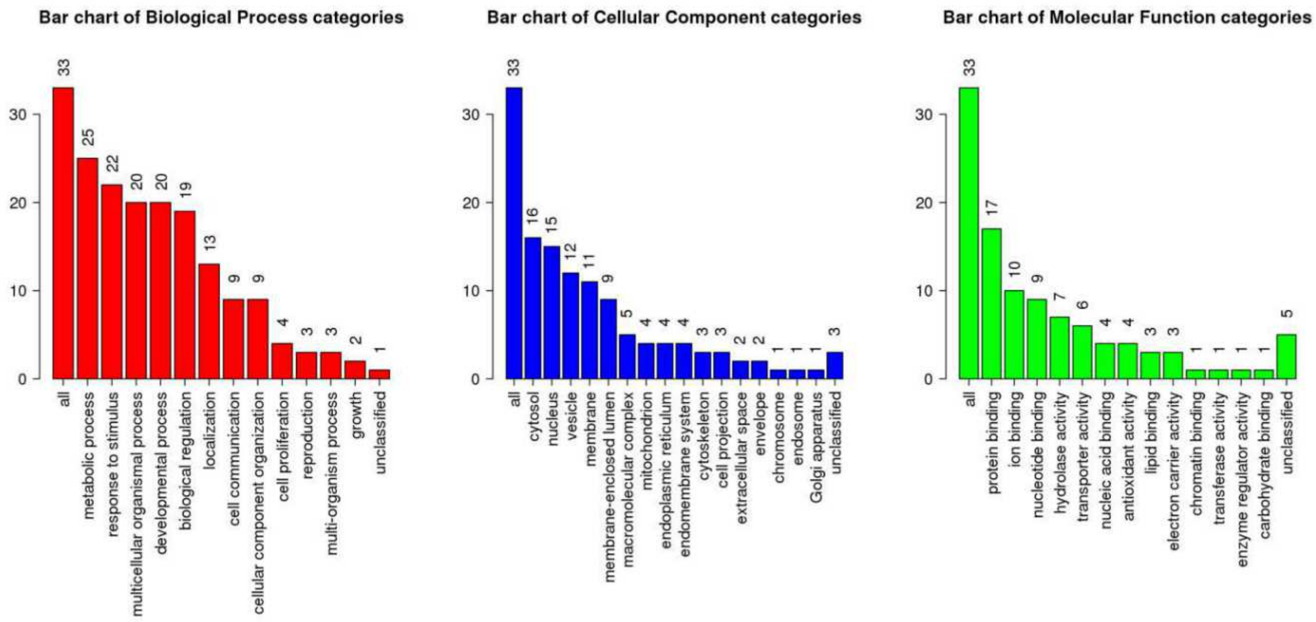

Figure S3. GO slim analysis of KMSGC by using WebGestalt

Table S1: Stratification LUAD tumors based on KEAP1 mutations

**Patients barcode**	**KEAP1 mutation status**
TCGA.05.4244	Wildtype
TCGA.05.4249	Wildtype
TCGA.05.4250	Wildtype
TCGA.05.4382	Wildtype
TCGA.05.4384	KEAP1 mutation
TCGA.05.4389	KEAP1 mutation
TCGA.05.4390	Wildtype
TCGA.05.4395	KEAP1 mutation
TCGA.05.4396	Wildtype
TCGA.05.4397	Wildtype
TCGA.05.4398	Wildtype
TCGA.05.4402	Wildtype
TCGA.05.4403	Wildtype
TCGA.05.4405	Wildtype
TCGA.05.4410	Wildtype
TCGA.05.4415	Wildtype
TCGA.05.4417	Wildtype
TCGA.05.4418	KEAP1 mutation
TCGA.05.4420	Wildtype
TCGA.05.4422	Wildtype
TCGA.05.4424	Wildtype
TCGA.05.4425	Wildtype
TCGA.05.4426	Wildtype
TCGA.05.4427	Wildtype
TCGA.05.4430	Wildtype
TCGA.05.4432	Wildtype
TCGA.05.4433	Wildtype
TCGA.05.4434	Wildtype
TCGA.05.5420	Wildtype
TCGA.05.5423	Wildtype
TCGA.05.5425	Wildtype
TCGA.05.5428	Wildtype
TCGA.05.5429	Wildtype
TCGA.05.5715	Wildtype
TCGA.35.4122	Wildtype
TCGA.35.4123	Wildtype
TCGA.35.5375	KEAP1 mutation

TCGA.38.4625	Wildtype
TCGA.38.4626	Wildtype
TCGA.38.4627	Wildtype
TCGA.38.4628	Wildtype
TCGA.38.4629	Wildtype
TCGA.38.4630	Wildtype
TCGA.38.4631	KEAP1 mutation
TCGA.38.4632	Wildtype
TCGA.38.6178	Wildtype
TCGA.38.7271	Wildtype
TCGA.38.A44F	Wildtype
TCGA.44.2656	Wildtype
TCGA.44.2657	Wildtype
TCGA.44.2661	Wildtype
TCGA.44.2665	Wildtype
TCGA.44.3396	Wildtype
TCGA.44.3398	Wildtype
TCGA.44.3918	Wildtype
TCGA.44.3919	Wildtype
TCGA.44.4112	Wildtype
TCGA.44.5643	Wildtype
TCGA.44.5644	Wildtype
TCGA.44.5645	Wildtype
TCGA.44.6145	Wildtype
TCGA.44.6146	Wildtype
TCGA.44.6147	Wildtype
TCGA.44.6148	Wildtype
TCGA.44.6774	Wildtype
TCGA.44.6775	Wildtype
TCGA.44.6776	KEAP1 mutation
TCGA.44.6777	Wildtype
TCGA.44.6778	KEAP1 mutation
TCGA.44.6779	KEAP1 mutation
TCGA.44.7659	Wildtype
TCGA.44.7660	KEAP1 mutation
TCGA.44.7661	Wildtype
TCGA.44.7662	Wildtype
TCGA.44.7667	KEAP1 mutation
TCGA.44.7669	KEAP1 mutation

TCGA.44.7670	Wildtype
TCGA.44.7671	KEAP1 mutation
TCGA.44.7672	Wildtype
TCGA.44.8117	Wildtype
TCGA.44.8119	Wildtype
TCGA.44.8120	Wildtype
TCGA.44.A479	Wildtype
TCGA.44.A47A	Wildtype
TCGA.44.A47B	Wildtype
TCGA.44.A47G	Wildtype
TCGA.44.A4SS	KEAP1 mutation
TCGA.44.A4SU	Wildtype
TCGA.49.4486	KEAP1 mutation
TCGA.49.4487	Wildtype
TCGA.49.4488	KEAP1 mutation
TCGA.49.4490	Wildtype
TCGA.49.4494	Wildtype
TCGA.49.4501	Wildtype
TCGA.49.4505	Wildtype
TCGA.49.4506	KEAP1 mutation
TCGA.49.4507	KEAP1 mutation
TCGA.49.4510	Wildtype
TCGA.49.4512	Wildtype
TCGA.49.4514	KEAP1 mutation
TCGA.49.6742	KEAP1 mutation
TCGA.49.6743	KEAP1 mutation
TCGA.49.6744	Wildtype
TCGA.49.6745	Wildtype
TCGA.49.6761	Wildtype
TCGA.49.6767	Wildtype
TCGA.50.5044	Wildtype
TCGA.50.5045	Wildtype
TCGA.50.5049	Wildtype
TCGA.50.5051	KEAP1 mutation
TCGA.50.5055	Wildtype
TCGA.50.5066	Wildtype
TCGA.50.5068	KEAP1 mutation
TCGA.50.5072	Wildtype
TCGA.50.5930	Wildtype

TCGA.50.5931	Wildtype
TCGA.50.5932	Wildtype
TCGA.50.5933	Wildtype
TCGA.50.5935	Wildtype
TCGA.50.5936	KEAP1 mutation
TCGA.50.5939	KEAP1 mutation
TCGA.50.5941	Wildtype
TCGA.50.5942	Wildtype
TCGA.50.5944	Wildtype
TCGA.50.5946	Wildtype
TCGA.50.6590	Wildtype
TCGA.50.6591	Wildtype
TCGA.50.6592	Wildtype
TCGA.50.6593	Wildtype
TCGA.50.6594	Wildtype
TCGA.50.6595	Wildtype
TCGA.50.6597	Wildtype
TCGA.50.6673	Wildtype
TCGA.50.7109	Wildtype
TCGA.50.8457	Wildtype
TCGA.50.8459	Wildtype
TCGA.50.8460	Wildtype
TCGA.53.7624	KEAP1 mutation
TCGA.53.7626	Wildtype
TCGA.53.7813	Wildtype
TCGA.53.A4EZ	KEAP1 mutation
TCGA.55.1592	Wildtype
TCGA.55.1595	Wildtype
TCGA.55.5899	KEAP1 mutation
TCGA.55.6543	Wildtype
TCGA.55.6642	Wildtype
TCGA.55.6712	Wildtype
TCGA.55.6968	Wildtype
TCGA.55.6969	Wildtype
TCGA.55.6970	KEAP1 mutation
TCGA.55.6971	Wildtype
TCGA.55.6972	KEAP1 mutation
TCGA.55.6975	Wildtype
TCGA.55.6978	Wildtype

TCGA.55.6979	Wildtype
TCGA.55.6980	Wildtype
TCGA.55.6981	Wildtype
TCGA.55.6982	Wildtype
TCGA.55.6983	Wildtype
TCGA.55.6984	Wildtype
TCGA.55.6985	Wildtype
TCGA.55.6986	Wildtype
TCGA.55.6987	KEAP1 mutation
TCGA.55.7227	Wildtype
TCGA.55.7281	Wildtype
TCGA.55.7283	Wildtype
TCGA.55.7284	Wildtype
TCGA.55.7570	Wildtype
TCGA.55.7573	Wildtype
TCGA.55.7574	Wildtype
TCGA.55.7576	Wildtype
TCGA.55.7724	Wildtype
TCGA.55.7725	Wildtype
TCGA.55.7726	Wildtype
TCGA.55.7727	Wildtype
TCGA.55.7728	Wildtype
TCGA.55.7815	KEAP1 mutation
TCGA.55.7816	Wildtype
TCGA.55.7903	Wildtype
TCGA.55.7907	Wildtype
TCGA.55.7910	Wildtype
TCGA.55.7911	Wildtype
TCGA.55.7913	Wildtype
TCGA.55.7914	Wildtype
TCGA.55.7994	Wildtype
TCGA.55.7995	KEAP1 mutation
TCGA.55.8085	KEAP1 mutation
TCGA.55.8087	Wildtype
TCGA.55.8089	Wildtype
TCGA.55.8090	Wildtype
TCGA.55.8091	Wildtype
TCGA.55.8092	Wildtype
TCGA.55.8094	KEAP1 mutation

TCGA.55.8096	Wildtype
TCGA.55.8097	Wildtype
TCGA.55.8203	Wildtype
TCGA.55.8204	Wildtype
TCGA.55.8205	Wildtype
TCGA.55.8206	Wildtype
TCGA.55.8207	Wildtype
TCGA.55.8208	Wildtype
TCGA.55.8299	Wildtype
TCGA.55.8301	Wildtype
TCGA.55.8302	KEAP1 mutation
TCGA.55.8505	KEAP1 mutation
TCGA.55.8506	KEAP1 mutation
TCGA.55.8507	Wildtype
TCGA.55.8508	Wildtype
TCGA.55.8510	KEAP1 mutation
TCGA.55.8511	Wildtype
TCGA.55.8512	Wildtype
TCGA.55.8513	Wildtype
TCGA.55.8514	Wildtype
TCGA.55.8614	Wildtype
TCGA.55.8615	Wildtype
TCGA.55.8616	Wildtype
TCGA.55.8619	Wildtype
TCGA.55.8620	KEAP1 mutation
TCGA.55.8621	Wildtype
TCGA.55.A48X	Wildtype
TCGA.55.A48Y	Wildtype
TCGA.55.A48Z	Wildtype
TCGA.55.A490	Wildtype
TCGA.55.A491	Wildtype
TCGA.55.A492	KEAP1 mutation
TCGA.55.A493	Wildtype
TCGA.55.A494	Wildtype
TCGA.55.A4DF	KEAP1 mutation
TCGA.55.A4DG	Wildtype
TCGA.62.8394	Wildtype
TCGA.62.8395	Wildtype
TCGA.62.8397	Wildtype

TCGA.62.8398	KEAP1 mutation
TCGA.62.8399	Wildtype
TCGA.62.8402	Wildtype
TCGA.62.A46O	KEAP1 mutation
TCGA.62.A46P	Wildtype
TCGA.62.A46R	Wildtype
TCGA.62.A46S	Wildtype
TCGA.62.A46U	Wildtype
TCGA.62.A46V	Wildtype
TCGA.62.A46Y	Wildtype
TCGA.62.A470	KEAP1 mutation
TCGA.62.A471	Wildtype
TCGA.62.A472	Wildtype
TCGA.64.1676	Wildtype
TCGA.64.1677	Wildtype
TCGA.64.1679	Wildtype
TCGA.64.1681	Wildtype
TCGA.64.5774	Wildtype
TCGA.64.5775	Wildtype
TCGA.64.5778	Wildtype
TCGA.64.5779	Wildtype
TCGA.64.5781	KEAP1 mutation
TCGA.64.5815	Wildtype
TCGA.67.3771	KEAP1 mutation
TCGA.67.3772	Wildtype
TCGA.67.3773	Wildtype
TCGA.67.4679	Wildtype
TCGA.67.6215	Wildtype
TCGA.67.6216	Wildtype
TCGA.67.6217	Wildtype
TCGA.69.7760	Wildtype
TCGA.69.7761	Wildtype
TCGA.69.7763	Wildtype
TCGA.69.7764	Wildtype
TCGA.69.7765	Wildtype
TCGA.69.7973	Wildtype
TCGA.69.7974	Wildtype
TCGA.69.7978	Wildtype
TCGA.69.7979	Wildtype

TCGA.69.7980	Wildtype
TCGA.69.8253	KEAP1 mutation
TCGA.69.8254	Wildtype
TCGA.69.8255	Wildtype
TCGA.69.A59K	Wildtype
TCGA.71.6725	Wildtype
TCGA.71.8520	Wildtype
TCGA.73.4658	Wildtype
TCGA.73.4659	Wildtype
TCGA.73.4662	Wildtype
TCGA.73.4666	KEAP1 mutation
TCGA.73.4668	Wildtype
TCGA.73.4670	KEAP1 mutation
TCGA.73.4675	Wildtype
TCGA.73.4676	KEAP1 mutation
TCGA.73.4677	KEAP1 mutation
TCGA.73.7498	KEAP1 mutation
TCGA.73.7499	Wildtype
TCGA.75.5122	Wildtype
TCGA.75.5125	Wildtype
TCGA.75.5126	Wildtype
TCGA.75.5146	Wildtype
TCGA.75.5147	Wildtype
TCGA.75.6203	Wildtype
TCGA.75.6205	Wildtype
TCGA.75.6206	KEAP1 mutation
TCGA.75.6207	Wildtype
TCGA.75.6211	KEAP1 mutation
TCGA.75.6212	Wildtype
TCGA.75.6214	Wildtype
TCGA.75.7025	Wildtype
TCGA.75.7027	KEAP1 mutation
TCGA.75.7030	Wildtype
TCGA.75.7031	Wildtype
TCGA.78.7143	Wildtype
TCGA.78.7145	Wildtype
TCGA.78.7146	KEAP1 mutation
TCGA.78.7147	Wildtype
TCGA.78.7148	Wildtype

TCGA.78.7149	KEAP1 mutation
TCGA.78.7150	Wildtype
TCGA.78.7152	Wildtype
TCGA.78.7153	KEAP1 mutation
TCGA.78.7154	KEAP1 mutation
TCGA.78.7155	Wildtype
TCGA.78.7156	KEAP1 mutation
TCGA.78.7158	KEAP1 mutation
TCGA.78.7159	Wildtype
TCGA.78.7160	Wildtype
TCGA.78.7161	Wildtype
TCGA.78.7162	KEAP1 mutation
TCGA.78.7163	Wildtype
TCGA.78.7166	KEAP1 mutation
TCGA.78.7167	KEAP1 mutation
TCGA.78.7220	Wildtype
TCGA.78.7535	Wildtype
TCGA.78.7536	Wildtype
TCGA.78.7537	Wildtype
TCGA.78.7539	Wildtype
TCGA.78.7540	Wildtype
TCGA.78.7542	Wildtype
TCGA.78.7633	KEAP1 mutation
TCGA.78.8640	Wildtype
TCGA.78.8648	Wildtype
TCGA.78.8655	Wildtype
TCGA.78.8660	Wildtype
TCGA.78.8662	Wildtype
TCGA.80.5607	Wildtype
TCGA.80.5608	Wildtype
TCGA.80.5611	Wildtype
TCGA.83.5908	Wildtype
TCGA.86.6562	Wildtype
TCGA.86.6851	Wildtype
TCGA.86.7701	Wildtype
TCGA.86.7711	Wildtype
TCGA.86.7713	Wildtype
TCGA.86.7714	Wildtype
TCGA.86.7953	Wildtype

TCGA.86.7954	Wildtype
TCGA.86.7955	Wildtype
TCGA.86.8054	Wildtype
TCGA.86.8055	Wildtype
TCGA.86.8056	Wildtype
TCGA.86.8073	Wildtype
TCGA.86.8074	Wildtype
TCGA.86.8075	Wildtype
TCGA.86.8076	Wildtype
TCGA.86.8278	Wildtype
TCGA.86.8279	Wildtype
TCGA.86.8280	Wildtype
TCGA.86.8281	KEAP1 mutation
TCGA.86.8358	KEAP1 mutation
TCGA.86.8359	Wildtype
TCGA.86.8585	Wildtype
TCGA.86.8668	Wildtype
TCGA.86.8669	Wildtype
TCGA.86.8671	Wildtype
TCGA.86.8672	Wildtype
TCGA.86.8673	Wildtype
TCGA.86.8674	KEAP1 mutation
TCGA.86.A456	Wildtype
TCGA.86.A4JF	Wildtype
TCGA.86.A4P7	Wildtype
TCGA.86.A4P8	Wildtype
TCGA.91.6828	Wildtype
TCGA.91.6829	Wildtype
TCGA.91.6830	Wildtype
TCGA.91.6831	Wildtype
TCGA.91.6835	Wildtype
TCGA.91.6836	Wildtype
TCGA.91.6840	Wildtype
TCGA.91.6847	Wildtype
TCGA.91.6848	KEAP1 mutation
TCGA.91.6849	KEAP1 mutation
TCGA.91.7771	Wildtype
TCGA.91.8496	Wildtype
TCGA.91.8497	Wildtype

TCGA.91.8499	Wildtype
TCGA.91.A4BC	Wildtype
TCGA.91.A4BD	Wildtype
TCGA.93.7347	Wildtype
TCGA.93.7348	Wildtype
TCGA.93.8067	Wildtype
TCGA.93.A4JN	Wildtype
TCGA.93.A4JO	Wildtype
TCGA.93.A4JP	Wildtype
TCGA.93.A4JQ	Wildtype
TCGA.95.7039	Wildtype
TCGA.95.7043	KEAP1 mutation
TCGA.95.7562	Wildtype
TCGA.95.7567	Wildtype
TCGA.95.7944	Wildtype
TCGA.95.7947	KEAP1 mutation
TCGA.95.7948	Wildtype
TCGA.95.8039	Wildtype
TCGA.95.8494	Wildtype
TCGA.95.A4VK	Wildtype
TCGA.95.A4VN	Wildtype
TCGA.95.A4VP	Wildtype
TCGA.97.7546	Wildtype
TCGA.97.7547	Wildtype
TCGA.97.7552	Wildtype
TCGA.97.7553	Wildtype
TCGA.97.7554	Wildtype
TCGA.97.7937	Wildtype
TCGA.97.7938	Wildtype
TCGA.97.7941	Wildtype
TCGA.97.8171	Wildtype
TCGA.97.8172	Wildtype
TCGA.97.8174	Wildtype
TCGA.97.8175	Wildtype
TCGA.97.8176	KEAP1 mutation
TCGA.97.8177	Wildtype
TCGA.97.8179	KEAP1 mutation
TCGA.97.8547	Wildtype
TCGA.97.8552	Wildtype

TCGA.97.A4LX	Wildtype
TCGA.97.A4M0	Wildtype
TCGA.97.A4M1	Wildtype
TCGA.97.A4M2	Wildtype
TCGA.97.A4M3	KEAP1 mutation
TCGA.97.A4M5	Wildtype
TCGA.97.A4M6	Wildtype
TCGA.97.A4M7	Wildtype
TCGA.99.7458	Wildtype
TCGA.99.8025	Wildtype
TCGA.99.8028	Wildtype
TCGA.99.8032	Wildtype
TCGA.99.8033	KEAP1 mutation
TCGA.J2.8192	Wildtype
TCGA.J2.8194	KEAP1 mutation
TCGA.J2.A4AD	Wildtype
TCGA.J2.A4AE	Wildtype
TCGA.J2.A4AG	Wildtype
TCGA.L4.A4E5	Wildtype
TCGA.L4.A4E6	Wildtype
TCGA.L9.A443	Wildtype
TCGA.L9.A444	Wildtype
TCGA.MN.A4N1	Wildtype
TCGA.MN.A4N4	Wildtype
TCGA.MN.A4N5	Wildtype
TCGA.MP.A4SV	Wildtype
TCGA.MP.A4SW	Wildtype
TCGA.MP.A4SY	Wildtype
TCGA.MP.A4T4	Wildtype
TCGA.MP.A4T6	Wildtype
TCGA.MP.A4T7	KEAP1 mutation
TCGA.MP.A4T8	Wildtype
TCGA.MP.A4T9	Wildtype
TCGA.MP.A4TA	Wildtype
TCGA.MP.A4TC	Wildtype
TCGA.MP.A4TD	Wildtype
TCGA.MP.A4TE	KEAP1 mutation
TCGA.MP.A4TF	Wildtype
TCGA.MP.A4TH	Wildtype

TCGA.MP.A4TI	Wildtype
TCGA.MP.A4TK	Wildtype
TCGA.MP.A5C7	KEAP1 mutation
TCGA.NJ.A4YF	Wildtype
TCGA.NJ.A4YG	Wildtype
TCGA.NJ.A4YI	Wildtype
TCGA.NJ.A4YP	Wildtype
TCGA.NJ.A4YQ	KEAP1 mutation
TCGA.NJ.A55A	Wildtype
TCGA.NJ.A55O	Wildtype
TCGA.NJ.A55R	KEAP1 mutation
TCGA.O1.A52J	Wildtype

Table S2: List of up and down regulated overlapping genes obtained from the DEG analysis of KEAP1 mutated *versus* WT TCGA LUAD tumors

Up regulated genes						
T-test			Wilcoxon test			Overlapping genes-33
Gene name	Fold Change	P-value	Gene name	Fold Change	P-value	AKR1C2
AKR1C2	6.22577199	6.22E-36	AKR1C2	7.973287	4.38E-31	AKR1C1
AKR1C1	5.27539217	1.12E-31	GPX2	6.594421	4.59E-25	GPX2
GPX2	5.18901554	9.72E-29	AKR1C1	6.14441	2.27E-30	ABCC2
ABCC2	3.46148392	6.19E-13	ABCC2	4.711354	1.84E-14	AKR1C3
AKR1C3	3.43028499	4.10E-31	UCHL1	4.013097	1.60E-20	CABYR
CABYR	3.02191006	1.47E-26	AKR1C3	3.829775	1.54E-29	UCHL1
UCHL1	2.98582679	6.73E-20	CABYR	3.128283	6.94E-27	TRIM16L
TRIM16L	2.88041915	4.17E-35	TRIM16L	3.120047	2.63E-34	S100P
S100P	2.84567569	2.83E-12	CPS1	3.08974	6.43E-05	CPS1
CPS1	2.60283699	7.50E-06	S100P	3.03509	4.07E-14	SLC7A11
SLC7A11	2.5799527	2.30E-29	SLC7A11	2.938262	5.92E-25	NQO1
NQO1	2.51708219	4.13E-33	NQO1	2.87386	7.96E-30	SRXN1
SRXN1	2.3574174	1.37E-32	SRXN1	2.691927	3.90E-32	OSGIN1
OSGIN1	2.34650288	4.32E-24	OSGIN1	2.648101	4.76E-28	TXNRD1
TXNRD1	2.15744664	1.91E-30	TXNRD1	2.568179	1.14E-28	GCLC
GCLC	2.02594655	3.91E-19	SLC7A2	2.497097	4.09E-08	CES1
CES1	2.01479231	2.03E-08	KYNU	2.479675	3.01E-10	PANX2
PANX2	2.00428378	8.95E-30	G6PD	2.396941	7.64E-26	G6PD
G6PD	1.93433109	1.46E-22	TSPAN7	2.375416	3.73E-15	TSPAN7
TSPAN7	1.92053673	6.55E-13	SLC16A14	2.210635	6.62E-11	CBR1
CBR1	1.91808199	3.49E-13	GCLC	2.198029	1.20E-22	CBX2
CBX2	1.86309965	3.43E-22	PANX2	2.162661	4.84E-26	KYNU
KYNU	1.81552807	6.07E-09	CES1	2.071875	1.67E-09	PIR
PIR	1.80576355	3.19E-31	SMOC1	2.052403	0.029573514	NEIL3
NEIL3	1.75752271	8.75E-16	PIR	2.04217	6.36E-28	UGDH
UGDH	1.73315733	8.45E-23	CBX2	2.011997	5.33E-23	PGD
PGD	1.72352446	1.83E-31	UGDH	2.005381	1.60E-24	CBR3
CBR3	1.7144523	1.30E-12	MAP2	2.00048	4.24E-13	SLC7A2
SLC7A2	1.66490371	3.08E-07	TRIM16	1.928616	3.17E-28	TRIM16
TRIM16	1.66402986	3.76E-28	ABP1	1.892875	0.012664322	ABCB6
ABCB6	1.56239919	5.58E-24	CARD14	1.831906	5.69E-11	GCLM
GCLM	1.55687797	2.33E-24	CBR1	1.825556	3.36E-18	SLC16A14
SLC16A14	1.51836805	2.25E-09	PGD	1.810587	4.61E-33	
			NEIL3	1.775105	1.57E-16	
			GSR	1.768656	1.88E-27	
			ABCB6	1.765827	1.88E-27	

RASD1	1.746061	3.72E-08
BTBD11	1.654456	1.02E-10
GCLM	1.653446	1.21E-26
CA12	1.636828	0.00062899
CBR3	1.571071	6.73E-16
PTGR1	1.542664	8.21E-22
SDCBP2	1.539571	4.67E-08
SLC16A4	1.528676	1.58E-05
CMBL	1.526846	2.05E-13
PDE4D	1.503486	1.44E-06

Down regulated genes								
T-test				Wilcoxon test				Overlapping genes-18
Gene name	Fold Change	P-value		Gene name	Fold Change	P-value		
SLC1A7	-2.83848361	1.12E-26		MFSD4	-2.81405	3.62268E-15		SLC1A7
MFSD4	-2.07753994	7.49E-16		SPTB	-2.45716	3.21694E-07		MFSD4
TFPI2	-2.05201566	6.05E-08		ITGB8	-2.43343	2.9137E-12		TFPI2
SYT12	-2.04680282	7.47E-11		VSIG1	-2.41088	1.96069E-09		SYT12
VSIG1	-2.02727732	1.96E-10		SLC1A7	-2.30125	1.85683E-16		VSIG1
DNAH11	-1.9951635	1.17E-11		DNAH11	-2.25243	4.04801E-12		DNAH11
LRRN4	-1.96372587	7.54E-11		TFPI2	-2.14113	1.76192E-08		LRRN4
ITGB8	-1.91106113	3.66E-11		CDH3	-2.12914	1.32474E-11		ITGB8
SLC34A2	-1.89286364	2.69E-09		RASGRF1	-2.11221	2.26878E-10		SLC34A2
CDH3	-1.86592525	2.25E-09		CX3CL1	-2.09048	4.07063E-18		CDH3
TNC	-1.81785732	1.51E-12		PNMAL1	-2.00915	6.30043E-07		TNC
HLA-DQB2	-1.75300661	1.10E-12		SLC34A2	-1.96616	4.6371E-12		HLA-DQB2
PIGR	-1.69879051	8.91E-07		DPP4	-1.94342	1.20195E-08		PIGR
ADORA1	-1.68597835	1.30E-12		LRRN4	-1.8957	2.71948E-11		ADORA1
RASGRF1	-1.67357752	4.18E-11		TNC	-1.85354	9.18543E-14		RASGRF1
CX3CL1	-1.66778141	5.83E-19		PIGR	-1.8277	1.73535E-07		CX3CL1
FCGBP	-1.60231062	4.52E-11		SYT12	-1.80663	3.74867E-10		FCGBP
HOXD1	-1.58549362	1.79E-09		RYR1	-1.80004	1.46321E-07		HOXD1
PLAT	-1.50994419	2.08E-21		WDR66	-1.79436	1.82765E-10		
ISM1	-1.50443224	1.35E-14		HOXD1	-1.78407	3.04136E-09		
				NMNAT2	-1.76644	2.64268E-06		
				PHYHD1	-1.76614	6.67837E-08		
				EFS	-1.71796	3.88067E-11		
				MT1E	-1.69422	1.2784E-09		
				STAC	-1.68343	8.12307E-09		
				TDRD10	-1.68115	1.43524E-05		
				ARHGAP20	-1.66822	7.6091E-13		

ADORA1	-1.65107	5.5017E-14
EYA2	-1.62576	6.62599E-06
SLC16A9	-1.62531	9.41522E-06
FCGBP	-1.6187	3.58409E-10
ROS1	-1.59788	9.29278E-09
ACHE	-1.59169	1.77681E-07
APLP1	-1.58662	2.23898E-05
VSTM2L	-1.58221	3.30746E-07
HLA-DQB2	-1.55569	1.11452E-12
KCNJ15	-1.53328	4.84457E-09
SEMA3A	-1.52598	1.70285E-07
CRABP2	-1.51135	9.76477E-06
ESPN	-1.50643	1.41822E-05

Table S4: Prediction of NRF2 binding sites of KMSGC by using the *in silico* analysis and NRF2-A549 cells ENCODE and Olagnier *et al*, 2018 (GSE113519) ChIP-Seq datas.

in silico analysis results					
**Chr**	**Start**	**End**	**Annotation**	**Distance to TSS**	**Gene Name**
chr2	220082669	220082680	intron (NM_005689, intron 1 of 18)	1064	ABCB6
chr10	101539728	101539739	Intergenic	-2621	ABCC2
chr10	101535777	101535788	Intergenic	-6572	ABCC2
chr10	101524554	101524565	Intergenic	-17795	ABCC2
chr10	101543924	101543935	intron (NM_000392, intron 1 of 31)	1575	ABCC2
chr10	4995798	4995809	Intergenic	-9650	AKR1C1
chr10	4999262	4999273	Intergenic	-6186	AKR1C1
chr10	5054973	5054984	intron (NM_001354, intron 1 of 10)	5246	AKR1C2
chr10	5114098	5114109	intron (NM_001253908, intron 1 of 8)	-22464	AKR1C3
chr10	5138029	5138040	intron (NM_003739, intron 1 of 8)	1467	AKR1C3
chr18	21772352	21772363	intron (NM_018030, intron 4 of 13)	52910	CABYR
chr18	21753406	21753417	intron (NM_018030, intron 7 of 13)	33964	CABYR
chr18	21727932	21727943	intron (NM_138644, intron 3 of 5)	8490	CABYR
chr18	21712935	21712946	3' UTR (NM_001135993, exon 14 of 14)	-5979	CABYR
chr18	21766008	21766019	intron (NM_018030, intron 4 of 13)	46566	CABYR
chr18	21773824	21773835	intron (NM_018030, intron 4 of 13)	54382	CABYR
chr17	77743996	77744007	Intergenic	-7975	CBX2
chr16	55852121	55852132	intron (NM_001025195, intron 7 of 13)	14948	CES1
chr16	55870438	55870449	Intergenic	-3369	CES1
chr16	55839857	55839868	intron (NM_001025195, intron 12 of 13)	27212	CES1
chr16	55868163	55868174	Intergenic	-1094	CES1
chr6	53413045	53413056	Intergenic	-3124	GCLC
chr6	53316051	53316062	Intergenic	93870	GCLC

chr6	53383078	53383089	intron (NM_001498, intron 3 of 15)	26843	GCLC
chr1	94470740	94470751	intron (NM_000350, intron 44 of 49)	-95592	GCLM
chr1	94430206	94430217	Intergenic	-55058	GCLM
chr1	94390951	94390962	Intergenic	-15803	GCLM
chr14	65421101	65421112	intron (NM_198686, intron 1 of 6)	-11484	GPX2
chr2	143348755	143348766	Intergenic	-286434	KYNU
chr2	143732013	143732024	intron (NM_001032998, intron 8 of 11)	96824	KYNU
chr2	143545067	143545078	Intergenic	-90122	KYNU
chr2	143591012	143591023	Intergenic	-44177	KYNU
chr2	143518234	143518245	Intergenic	-116955	KYNU
chr2	143442928	143442939	Intergenic	-192261	KYNU
chr2	143276473	143276484	Intergenic	-358716	KYNU
chr2	143560938	143560949	Intergenic	-74251	KYNU
chr2	143680029	143680040	intron (NM_001032998, intron 3 of 11)	44840	KYNU
chr2	143734293	143734304	intron (NM_001032998, intron 8 of 11)	99104	KYNU
chr4	178219158	178219169	Intergenic	-11827	NEIL3
chr4	178128918	178128929	Intergenic	-102067	NEIL3
chr4	178296772	178296783	Intergenic	65787	NEIL3
chr4	178293902	178293913	Intergenic	62917	NEIL3
chr4	178071979	178071990	Intergenic	-159006	NEIL3
chr4	178128313	178128324	Intergenic	-102672	NEIL3
chr16	69760908	69760919	promoter-TSS (NM_001286137)	-343	NQO1
chr16	69768969	69768980	Intergenic	-8404	NQO1
chr16	83976952	83976963	Intergenic	-9869	OSGIN1
chr1	10440740	10440751	3' UTR (NM_015074, exon 47 of 47)	-18303	PGD
chrX	15514662	15514673	Intergenic	-2957	PIR
chr2	230964071	230964082	Intergenic	-30362	SLC16A14
chr2	230934484	230934495	promoter-TSS (NM_152527)	-775	SLC16A14
chr2	230984820	230984831	Intergenic	-51111	SLC16A14
chr2	230861341	230861352	intron (NM_174899, intron 2 of 3)	72368	SLC16A14

chr2	230974017	230974028	Intergenic	-40308	SLC16A14
chr2	230973814	230973825	Intergenic	-40105	SLC16A14
chr2	230983545	230983556	Intergenic	-49836	SLC16A14
chr4	139173880	139173891	Intergenic	-10383	SLC7A11
chr4	139115227	139115238	intron (NM_014331, intron 6 of 11)	48270	SLC7A11
chr4	139156848	139156859	intron (NM_014331, intron 2 of 11)	6649	SLC7A11
chr8	17318002	17318013	Intergenic	-36589	SLC7A2
chr8	17357190	17357201	intron (NM_001008539, intron 1 of 11)	2599	SLC7A2
chr20	641249	641260	TTS (NM_033129)	-7241	SRXN1
chr17	18633473	18633484	intron (NM_001353225, intron 2 of 3)	8143	TRIM16L
chrX	38529382	38529393	intron (NM_004615, intron 2 of 7)	108657	TSPAN7
chrX	38525780	38525791	intron (NM_004615, intron 2 of 7)	105055	TSPAN7
chrX	38376403	38376414	Intergenic	-44322	TSPAN7
chrX	38423767	38423778	intron (NM_004615, intron 1 of 7)	3042	TSPAN7
chrX	38353468	38353479	Intergenic	-67257	TSPAN7
chrX	38492848	38492859	intron (NM_004615, intron 1 of 7)	72123	TSPAN7
chrX	38319262	38319273	Intergenic	-101463	TSPAN7
chrX	38512599	38512610	intron (NM_004615, intron 1 of 7)	91874	TSPAN7
chrX	38379982	38379993	Intergenic	-40743	TSPAN7
chr12	104613290	104613301	intron (NM_001093771, intron 1 of 16)	3739	TXNRD1
chr12	104667784	104667795	intron (NM_001093771, intron 3 of 16)	-12670	TXNRD1
chr12	104674045	104674056	intron (NM_001093771, intron 3 of 16)	-6409	TXNRD1
chr4	41259458	41259469	promoter-TSS (NR_102709)	566	UCHL1
chr4	39516837	39516848	intron (NM_001184701, intron 1 of 10)	12375	UGDH

ENCODE-NRF2-A549 ChIP-Seq results

**Chr**	**Start**	**End**	**Annotation**	**Distance to TSS**	**Gene Name**
chr2	220082581	220082774	intron (NM_005689, intron 1 of 18)	1061	ABCB6
chr10	101544969	101545111	intron (NM_000392, intron 2 of 31)	2685	ABCC2

chr10	101520086	101520356	Intergenic	-22134	ABCC2
chr10	101535727	101535906	Intergenic	-6538	ABCC2
chr10	101594148	101594418	exon (NM_000392, exon 24 of 32)	51928	ABCC2
chr10	4995662	4995932	Intergenic	-9657	AKR1C1
chr10	4983820	4984003	Intergenic	-21542	AKR1C1
chr10	4989958	4990228	Intergenic	-15361	AKR1C1
chr10	5062507	5062690	Intergenic	-2374	AKR1C2
chr10	5051568	5051669	intron (NM_001354, intron 1 of 10)	-5413	AKR1C2
chr10	5075630	5075819	Intergenic	-15233	AKR1C3
chr10	5137902	5138172	intron (NM_003739, intron 1 of 8)	1469	AKR1C3
chr10	5113969	5114237	intron (NM_001253908, intron 1 of 8)	-22465	AKR1C3
chr10	5121057	5121327	intron (NM_001253908, intron 1 of 8)	-15376	AKR1C3
chr18	21754086	21754356	intron (NM_018030, intron 7 of 13)	34773	CABYR
chr18	21717118	21717388	Intergenic	-1667	CABYR
chr21	37442018	37442288	promoter-TSS (NM_001757)	-69	CBR1
chr17	77743865	77744135	Intergenic	-7977	CBX2
chr17	77738642	77738912	Intergenic	-13200	CBX2
chr16	55870288	55870558	Intergenic	-3348	CES1
chr6	53412985	53413121	Intergenic	-3126	GCLC
chr1	94374911	94375147	5' UTR (NM_002061, exon 1 of 7)	125	GCLM
chr1	94429335	94429424	Intergenic	-54226	GCLM
chr14	65409431	65409701	promoter-TSS (NM_002083)	57	GPX2
chr2	143630978	143631187	Intergenic	-4112	KYNU
chr2	143632017	143632287	Intergenic	-3043	KYNU
chr4	178229533	178229613	Intergenic	-1418	NEIL3
chr16	69760750	69761049	promoter-TSS (NM_001286137)	-329	NQO1
chr16	83986669	83986804	promoter-TSS (NM_182981)	-90	OSGIN1
chr22	50608460	50608730	promoter-TSS (NR_027691)	-565	PANX2
chr1	10448166	10448350	Intergenic	-10791	PGD
chr1	10447672	10447942	Intergenic	-11242	PGD

chrX	15511347	15511489	5' UTR (NM_003662, exon 1 of 10)	293	PIR
chr4	139163559	139163670	promoter-TSS (NM_014331)	-112	SLC7A11
chr4	139173736	139174006	Intergenic	-10368	SLC7A11
chr4	139156747	139156944	intron (NM_014331, intron 2 of 11)	6657	SLC7A11
chr20	633997	634297	promoter-TSS (NM_080725)	-133	SRXN1
chrX	38429702	38429972	intron (NM_004615, intron 1 of 7)	9106	TSPAN7
chr12	104613165	104613452	intron (NM_001093771, intron 1 of 16)	3752	TXNRD1
chr12	104682531	104682641	intron (NM_001261445, intron 1 of 14)	2126	TXNRD1
chr12	104680586	104680849	5' UTR (NM_001261445, exon 1 of 15)	258	TXNRD1
chr12	104671170	104671440	intron (NM_001093771, intron 3 of 16)	-9155	TXNRD1
chr12	104680129	104680399	promoter-TSS (NM_001261445)	-196	TXNRD1
chr4	41259304	41259574	promoter-TSS (NR_102709)	541	UCHL1
chr4	39516751	39517052	intron (NM_001184701, intron 1 of 10)	12316	UGDH

**Olagnier et al, 2018 ChIP-Seq results**					
Chr	Start	End	Dist to Start	Position	Gene List
10	101,535,606	101,536,203	-6,655	upstream	ABCC2
10	101,539,576	101,540,297	-2,367	upstream	ABCC2
10	101,543,344	101,545,578	2,561	in gene	ABCC2
10	101,594,080	101,594,553	51,777	in gene	ABCC2
10	4,998,865	4,999,628	-6,158	upstream	AKR1C1
10	5,051,234	5,052,009	8,719	in gene	AKR1C2
10	5,052,669	5,052,882	7,439	in gene	AKR1C2
10	5,061,156	5,061,353	-1,105	upstream	AKR1C2
10	5,061,822	5,063,377	-2,337	upstream	AKR1C2
10	5,135,177	5,135,517	-1,272	upstream	AKR1C3
10	5,137,996	5,138,593	1,560	in gene	AKR1C3
17	77,743,096	77,744,490	-7,945	upstream	CBX2
2	211,413,724	211,414,001	71,479	in gene	CPS1
6	53,409,665	53,409,992	-25	upstream	GCLC

6	53,412,707	53,413,638		-3,193	upstream	GCLC
1	94,373,936	94,375,444		36	in gene	GCLM
2	143,625,855	143,626,160		-9,211	upstream	KYNU
2	143,628,268	143,628,521		-6,811	upstream	KYNU
2	143,630,925	143,632,304		-4,091	upstream	KYNU
4	178,229,311	178,229,741		-1,487	upstream	NEIL3
16	69,760,128	69,762,066		-363	upstream	NQO1
16	83,985,898	83,987,573		4,016	in gene	OSGIN1
1	10,471,540	10,471,741		12,579	in gene	PGD
1	10,474,785	10,475,941		16,547	in gene	PGD
1	10,476,216	10,476,438		17,187	in gene	PGD
1	10,476,527	10,476,830		17,603	in gene	PGD
4	139,143,256	139,143,579		20,095	in gene	SLC7A11
4	139,156,581	139,157,505		6,639	in gene	SLC7A11
4	139,163,119	139,163,744		-17	upstream	SLC7A11
17	18,625,091	18,625,407		-186	upstream	TRIM16L
17	18,626,900	18,627,142		1,606	in gene	TRIM16L
12	104,609,420	104,610,033		9	in gene	TXNRD1
12	104,612,631	104,614,373		3,785	in gene	TXNRD1
12	104,670,612	104,671,587		61,801	in gene	TXNRD1
12	104,679,576	104,683,538		71,145	in gene	TXNRD1
4	41,258,754	41,260,230		462	in gene	UCHL1
4	39,516,284	39,517,062		12,386	in gene	UGDH
4	39,517,292	39,517,928		11,762	in gene	UGDH
4	39,528,889	39,529,166		258	in gene	UGDH
4	39,529,245	39,529,710		-254	upstream	UGDH

Table S5: Fold change values of KMSGC obtained from the RNA-Seq data of NRF2 knockdown lung cancer cell lines

**KMSGC expression in H2122 cells after NRF2 knockdown- Bar-Peled *et al,* 2017 (GSE89569)**	
**Gene**	**Fold change (NRF2 KD vs. Control)**
AKR1C1	-3.31
CBR3	-3.17
NEIL3	-3.14
GCLM	-3.01
TRIM16L	-2.88
UCHL1	-2.75
OSGIN1	-2.69
SRXN1	-2.50
CBR1	-2.46
CABYR	-2.43
CBX2	-2.43
UGDH	-2.38
TSPAN7	-2.36
PIR	-2.35
ABCB6	-2.20
TXNRD1	-2.05
PANX2	-1.89
ABCC2	-1.86
GPX2	-1.69
CES1	-1.45

**KMSGC expression in A549 cells after NRF2 knockdown- Olagnier *et al,* 2018 (GSE113519)**	
**Gene**	**Fold change (NRF2 KD vs. Control)**
TXNRD1	-3.6
TRIM16L	-3.5
SLC7A11	-3.45
OSGIN1	-3.21
GCLM	-2.82
G6PD	-2.56
AKR1C1	-2.51
PANX2	-2.48
SRXN1	-2.38
TRIM16	-2.37
UGDH	-2.29
AKR1C2	-2.22
GCLC	-2.16
NQO1	-2.07
TSPAN7	-2.07
ABCC2	-2.01
AKR1C3	-1.84
PGD	-1.69
NEIL3	-1.68
ABCB6	-1.63

GCLC	-1.40
G6PD	-1.35
AKR1C3	-1.10
NQO1	-1.03
CPS1	-0.94
SLC7A11	-0.75
TRIM16	-0.62
PGD	-0.59
AKR1C2	-0.57
SLC16A14	-0.49
SLC7A2	-0.34
KYNU	-0.10
S100P	0.45

CES1	-1.61
CBR3	-1.46
KYNU	-1.45
CBR1	-1.43
UCHL1	-1.38
PIR	-1.31
GPX2	-0.98
CABYR	-0.82
CBX2	-0.27
S100P	-0.12
CPS1	0.12
SLC7A2	0.37
SLC16A14	0.5

Table S6: List of primers used in this study for the qRT-PCR analysis

mRNA	Function	Sequence
CBX2	Forward	5′-AGTGCATCCTGAGCAAGCG-3′
	Reverse	5′-TCCCAGCTGTTATGTTTGGAGG-3′
G6PD	Forward	5′-TGACCTGGCCAAGAAGAAGA-3′
	Reverse	5′-CAAAGAAGTCCTCCAGCTTG-3′
AKR1C1	Forward	5′-CGAGAAGAACCATGGGTGGA-3′
	Reverse	5′-GGCACAAAGGACTGGGTCC-3′
NQO1	Forward	5′-GGAGAGTTTGCTTACACTTACGC-3′
	Reverse	5′-AGTGGTGATGGAAAGCACTGCCTTC-3′
TRM16L	Forward	5′-GTCCTTCTGTGTGCATTGGG-3′
	Reverse	5′-TCTGACACCGACACCAGAAC-3′
NEIL3	Forward	5′-CAAGCGTCCTAATTGTGGTCA-3′
	Reverse	5′-CCCTGCTAGATGTCCAACTGATT-3′
UCHL1	Forward	5′-CCTGTGGCACAATCGGACTTA-3′
	Reverse	5′-CATCTACCCGACATTGGCCTT-3′
TSPAN7	Forward	5′-ACCAAACCTGTGATAACCTGTCT-3′
	Reverse	5′-AGGGAGATATAGGTGCCCAGA-3′
